# Rhodopsin in the Dark Hot Sea: Molecular Analysis of Rhodopsin in a Snailfish, *Careproctus rhodomelas*, Living near the Deep-Sea Hydrothermal Vent

**DOI:** 10.1371/journal.pone.0135888

**Published:** 2015-08-14

**Authors:** Rie Sakata, Ryo Kabutomori, Keiko Okano, Hiromasa Mitsui, Akihiro Takemura, Tetsuya Miwa, Hiroyuki Yamamoto, Toshiyuki Okano

**Affiliations:** 1 Department of Electrical Engineering and Bioscience, Graduate School of Advanced Science and Engineering, Waseda University (TWIns), Tokyo, Japan; 2 Department of Chemistry, Biology, and Marine Science, Faculty of Science, University of the Ryukyus, Okinawa, Japan; 3 Marine Technology Development Department, Marine Technology and Engineering Center, Japan Agency for Marine Earth Science and Technology (JAMSTEC), Yokosuka, Japan; 4 Environmental Impact Assessment Research Group, Research and Development Centre for Submarine Resources, Japan Agency for Marine Earth Science and Technology (JAMSTEC), Yokosuka, Japan; University of Western Australia, AUSTRALIA

## Abstract

Visual systems in deep-sea fishes have been previously studied from a photobiological aspect; however, those of deep-sea fish inhabiting the hydrothermal vents are far less understood due to sampling difficulties. In this study, we analyzed the visual pigment of a deep-sea snailfish, *Careproctus rhodomelas*, discovered and collected only near the hydrothermal vents of oceans around Japan. Proteins were solubilized from the *C*. *rhodomelas* eyeball and subjected to spectroscopic analysis, which revealed the presence of a pigment characterized by an absorption maximum (λ_max_) at 480 nm. Immunoblot analysis of the ocular protein showed a rhodopsin-like immunoreactivity. We also isolated a retinal cDNA encoding the entire coding sequence of putative *C*. *rhodomelas* rhodopsin (CrRh). HEK293EBNA cells were transfected with the CrRh cDNA and the proteins extracted from the cells were subjected to spectroscopic analysis. The recombinant CrRh showed the absorption maximum at 480 nm in the presence of 11-*cis* retinal. Comparison of the results from the eyeball extract and the recombinant CrRh strongly suggests that CrRh has an A_1_-based 11-*cis*-retinal chromophore and works as a photoreceptor in the *C*. *rhodomelas* retina, and hence that *C*. *rhodomelas* responds to dim blue light much the same as other deep-sea fishes. Because hydrothermal vent is a huge supply of viable food, *C*. *rhodomelas* likely do not need to participate diel vertical migration and may recognize the bioluminescence produced by aquatic animals living near the hydrothermal vents.

## Introduction

Photoreceptors in rods and cones are comprised of rhodopsins and cone visual pigments, and these visual pigments are composed of apoprotein (opsin) and 11-*cis*-retinal chromophore. Rods are more sensitive to light than cones, but the light response of rods is slower than that of cones [1]. The visual system in animals adapts to the environment by changing the structures of both visual cells and photoreceptor pigment proteins [2–4], and therefore, sequencing and characterization of the photoreceptors of animals living in unusual visual environments would be a fruitful approach towards understanding the mechanism underlying such diversified photoreception systems.

The deep ocean is a unique photic environment because of the extraordinarily low light levels present, which places different evolutionary constraints on the visual system of deep-sea fish. There are two main natural sources of illumination in the deep sea: residual sunlight and bioluminescence. Due to the spectral filtering of water, the photic environment at depth primarily consists of a narrow band of radiation between 470 and 480 nm [5], or a dim blue light. The second source of light in the deep sea is the bioluminescence produced by the aquatic animals themselves, whose peak emission is usually around the same wavelengths as the remaining sunlight [6, 7].

In general, light penetrating into the deep sea is not enough for photosynthesis, so there is no phytoplankton-based ecosystem. Organisms can be found in relatively high abundances in the deep sea only around hot and cold seep sites such as near hydrothermal vents. Hydrothermal vents are fissures that issue high-temperature water from the seabed, and physicochemical environment and factors such as light conditions differ from the usual deep sea. The vent provides a unique ecosystem, in which chemosynthetic bacteria around the vents form the base of the food chain, and there are many peculiar animals not found in deep-sea areas absent of vents [8, 9].

Many studies including spectroscopic analyses were conducted on visual pigments in deep-sea and deep-freshwater animal species and investigated the evolution of their unique photoreception systems [10–20]. One example is Lake Baikal cottoids, rhodopsins of which show adaptive evolution that their spectral properties have been blue-shifted as to most efficiently absorb the light under their living environments [14]. However, little is known about the biology and visual systems of deep-sea fish endemic to the hydrothermal vent. Hydrothermal vents are reported to generate light in the near infrared spectrum [17], and some crustacean species are suggested to use this as a light source [17–20]. In this study, we aimed to understand the photoreception system in fish living near the hydrothermal vents by analyzing their visual pigment that might be red-shifted to receive the infrared radiation. We selected a deep-sea snailfish, *Careproctus rhodomelas*, which has been discovered near the hydrothermal vents in the oceans around Japan [21, 22]. There are only a few literatures treating this animal [23], and we have no knowledge on its visual system. Here we report that CrRh has characteristics typical of deep-sea fish living distant from the hydrothermal vents; *i*.*e*. the same blue-shifted spectral properties.

## Materials and Methods

### Animals

All studies were approved by the Committee for Animal Experimentation at the School of Science and Engineering at Waseda University (permission # 08A04). *C*. *rhodomelas* was collected from active hydrothermal vent field on the Hatoma Knoll (24°51’N; 123°50’E; depth 1480–1530 m) of the Okinawa Trough, Japan. The dive expedition for sampling using a remotely-operated vehicle (ROV) Hyper-Dolphin equipped with Deep Aquarium system, which is a sampling tool to hold *in situ* condition during operation, was conducted in a research cruise (NT08-15) of R/V Natsushima [22]. The Deep Aquarium system enabled to keep the snailfish alive without decompression-induced damage during ROV operation. We confirmed that no specific permissions were required for these locations/activities. We confirmed the present field study did not involve endangered or protected species. To minimize suffering, the spinal cord was rapidly cut under anesthesia on ice, and the eyeballs were collected from *C*. *rhodomelas*. One eyeball was kept in RNA*later* (Ambion) at -80°C for RNA extraction, and the other was frozen in the dark at -80°C for protein extraction.

### Extraction and spectroscopic analysis of *C*. *rhodomelas* ocular pigments

All extraction and spectroscopic analysis experiments were performed under dim red light (>640 nm, ~120 μW/cm^2^). A frozen *C*. *rhodomelas* eyeball was incubated in the dark at 4°C for 10 min and homogenized with a dounce homogenizer (25 strokes) in buffer P [50 mM HEPES-NaOH, 140 mM NaCl, 1 mM DTT (pH 6.6), 50 ml/tablet Complete (Roche), 1% (v/v) CHAPS]. The homogenate was centrifuged at 50,000 g for 20 min, and the clear supernatant was collected as the first extract. Until the supernatant became colorless, this extraction procedure was repeated three times to obtain a total of four extracts.

The optical cell sample (light path, 1 cm) was kept at 28°C during the spectroscopic analysis. After an initial scan of the extract from 250 nm to 800 nm, hydroxylamine (NH_2_OH) was added to the extract to a final concentration of 100 mM for complete conversion of residual light-activated rhodopsin into retinal-oxime and opsin, and then the absorption spectra were recorded several times until the spectrum showed no significant change. Then, the residual light-absorbing materials in the sample were bleached by successive irradiations with orange (O564 filter, HOYA) and yellow (Y522 filter, HOYA) light. All four extracts were analyzed to estimate the total amount of rhodopsin in the *C*. *rhodomelas* eyeball.

### Cloning of cDNA for *CrRh*

Total RNA was isolated from the *C*. *rhodomelas* eyeball using TRIzol Reagent (Life Technologies), and first-strand cDNAs were synthesized with SuperScript III reverse transcriptase (Life Technologies) using oligo(dT)_21_ primer or KSII(dT)_21_ (5’- GAGGT CGACG GTATC GATAA GCTTT TTTTT TTTTT TTTTT TTT -3’) primer. The *CrRh* cDNA fragments were amplified using *Taq* polymerase (Roche) and a pair of degenerate primers (df_rhoF1, 5’- TAYCC HCAGT AYTAC CTKG -3’ and df_rhoR2, 5’- GAACT GYYTG TTCAW SMARA TGTAG -3’) that were designed based on a conserved region of rhodopsin genes from the species *Oryzias latipes*, *Takifugu rubripes*, *Xenopus tropicalis*, *Gallus gallus*, and *Bos taurus*. Complementary DNAs including 5’- and 3’- UTRs were obtained using RACE. A pair of PCR primers (CrRh-full-F, 5’- GTCCG TCTCC ATCAC TCTCC GGAAG -3’ and CrRh-full-R, 5’- CTGTA GACGT TAGCC TTCAG TCGTT TC -3’) was designed in 5’- and 3’- UTRs to amplify cDNA fragments covering entire coding sequences with *PfuUltra* (Stratagene). The cDNA fragments were inserted into the pMD20-T vector (TaKaRa), and at least five independent clones were sequenced to obtain a full-length cDNA clone without presumed PCR errors.

### Immunoblot analysis

SDS-PAGE, Coomassie brilliant blue (CBB) stain, and western blotting were performed as described previously [24]. The blots were treated with a blocking solution of 1% skim milk in TBS [50 mM Tris, 200 mM NaCl, 1 mM MgCl_2_ (pH 7.4)] for 1h at 37°C, and then incubated overnight at 4°C with the primary antibody (anti-chicken rhodopsin antibodies; cRh-N, cRh-C, [25]; anti-toad rhodopsin antiserum; ToadRh-AS, [24]) diluted in the blocking solution. After washing three times for 5 min each with the blocking solution at room temperature, the blots were successively incubated with an alkaline phosphatase-labeled anti-mouse IgG antibody (1 mg/ml, Cell Signaling Technology: #7056) in the blocking solution and washed three times with TBS. The signals were visualized using CDP-Star Reagent (New England Biolabs) and detected with X-ray photographic film (Amersham Hyperfilm ECL, GE Healthcare).

### Expression and spectroscopy of recombinant CrRh

CrRh cDNA was cloned into pREP4 mammalian expression vector (Life Technologies) and transfected into HEK293EBNA by the calcium-phosphate method [26]. Forty-eight hours after the transfection, the cells were collected and homogenized with buffer P-10 (50 mM HEPES, 10 mM NaCl, 1 mM DTT, 4 μg/ml aprotinin and 4 μg/ml leupeptin). The homogenate was centrifuged at 101,000 *g* for 15 min at 4°C. To reconstitute the CrRh, the pellet was suspended in the buffer P-10 containing 1% CHAPS and 0.2 OD 11-*cis* retinal (vitamin A_1_-retinal). To perform photobleaching of CrRh, one molar NH_2_OH (Hydroxylamine hydrochloride; pH 6.6 adjusted with NaOH) was added to the final concentration of 50 mM. Then the sample was sequentially irradiated with green LED (501 μW/cm^2^; OSTCXBC1C1S OptoSupply; λ_max_ = 520 nm; λ_50%_ = 485 nm, 555 nm; λ_1%_ = 463 nm, 608 nm) for totally 1 min.

## Results

### Absorbance spectrum of *C*. *rhodomelas* visual pigment

**Fig 1:**
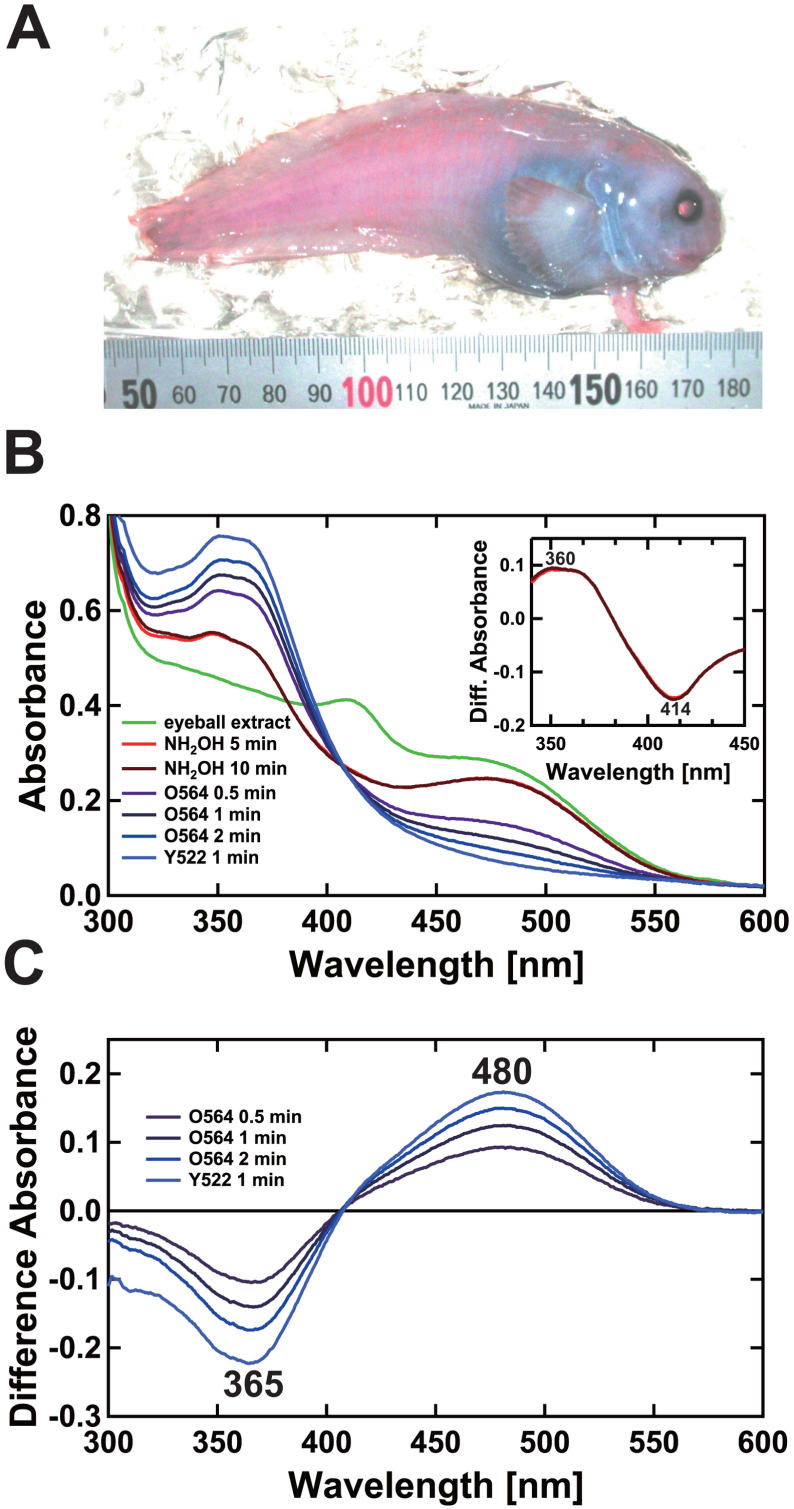
Absorbance changes of *C*. *rhodomelas* eyeball extract before and after NH_2_OH addition and subsequent light irradiation. (A) Order Scorpaeniformes, Liparidae, snailfish. This snailfish is rosy and flexible like gelatin. They inhabit hydrothermal vent areas at depths of approximately 1500 m. The body length and eyeball diameter of the fish shown above were 127 mm and 7 mm, respectively. (B) Absorption spectra of the detergent extract from a *C*. *rhodomelas* eyeball with buffer P containing 1% CHAPS (curve "eyeball extract" in panel B). One molar NH_2_OH was added to a final concentration of 100 mM (curves "NH_2_OH 5 min" and "NH_2_OH 10 min"). The sample was then sequentially irradiated with orange light using an O564 filter (8 mW/cm^2^, λ_T50%_ = 564 nm, λ_T0.1%_ = 540 nm) for a total of 2 min (curves "O564 0.5 min", "O564 1 min" and "O564 2 min") and yellow light using a Y522 filter (24 mW/cm^2^, λ_T50%_ = 522 nm, λ_T0.1%_ = 501 nm) for a total of 1 min (curve "Y522 1 min"). Inset shows difference absorbance spectra before and after the addition of NH_2_OH. (C) Difference absorbance spectra before the first irradiation and after each irradiation are shown.

The extract obtained from eyeball of *C*. *rhodomelas* (Fig 1A) was subjected to photospectroscopic analysis and the absolute absorption spectrum showed the presence of component(s) with absorption around 410 nm and 480 nm (Fig 1B, "eyeball extract"). To more precisely examine the presence of rhodopsin, we added NH_2_OH to the extract until a final concentration of 100 mM was reached, thereby converting free 11-*cis* retinal and cone opsin chromophore retinals into retinal oximes [27] generally without affecting the rhodopsins, with the exception of lamprey rhodopsin [28]. The peak around 410 nm rapidly decreased along with the production of a compound having an absorption in the UV region (around 360 nm; Fig 1B, inset). Difference absorption spectra showed a peak at 414 nm for the decayed compound(s) (tentatively termed P_414_, Fig 1B, inset). It was difficult to infer the identity of P_414_ on the basis of the spectral properties. On the other hand, the absorption appearing around 480 nm may be accounted for by rhodopsin.

After the NH_2_OH bleaching was completed (Fig 1B, "NH_2_OH 10 min"), the sample was irradiated successively with orange light and yellow light until the spectrum showed no significant change. The difference spectra between the absorption spectra before the first irradiation (Fig 1B, "NH_2_OH 10 min") and after each irradiation (Fig 1B, "O564 0.5 min", "O564 1 min", "O564 2 min", "Y522 1 min") formed an isosbestic point around 410 nm and also showed the same difference absorption maximum (480 nm; Fig 1C). These results strongly suggest that the observed spectral change was due to a photoreaction from a presumed rhodopsin to opsin and retinal oxime. As seen in the chicken rhodopsin [27], CrRh likely bleaches with light into opsin and all-*trans* retinal through photointermediates, the chromophore of which are converted into the retinal oxime in the presence of 100 mM NH_2_OH (Fig 1B).

### Isolation of cDNA for CrRh and its phylogenetic analysis

**Fig 2:**
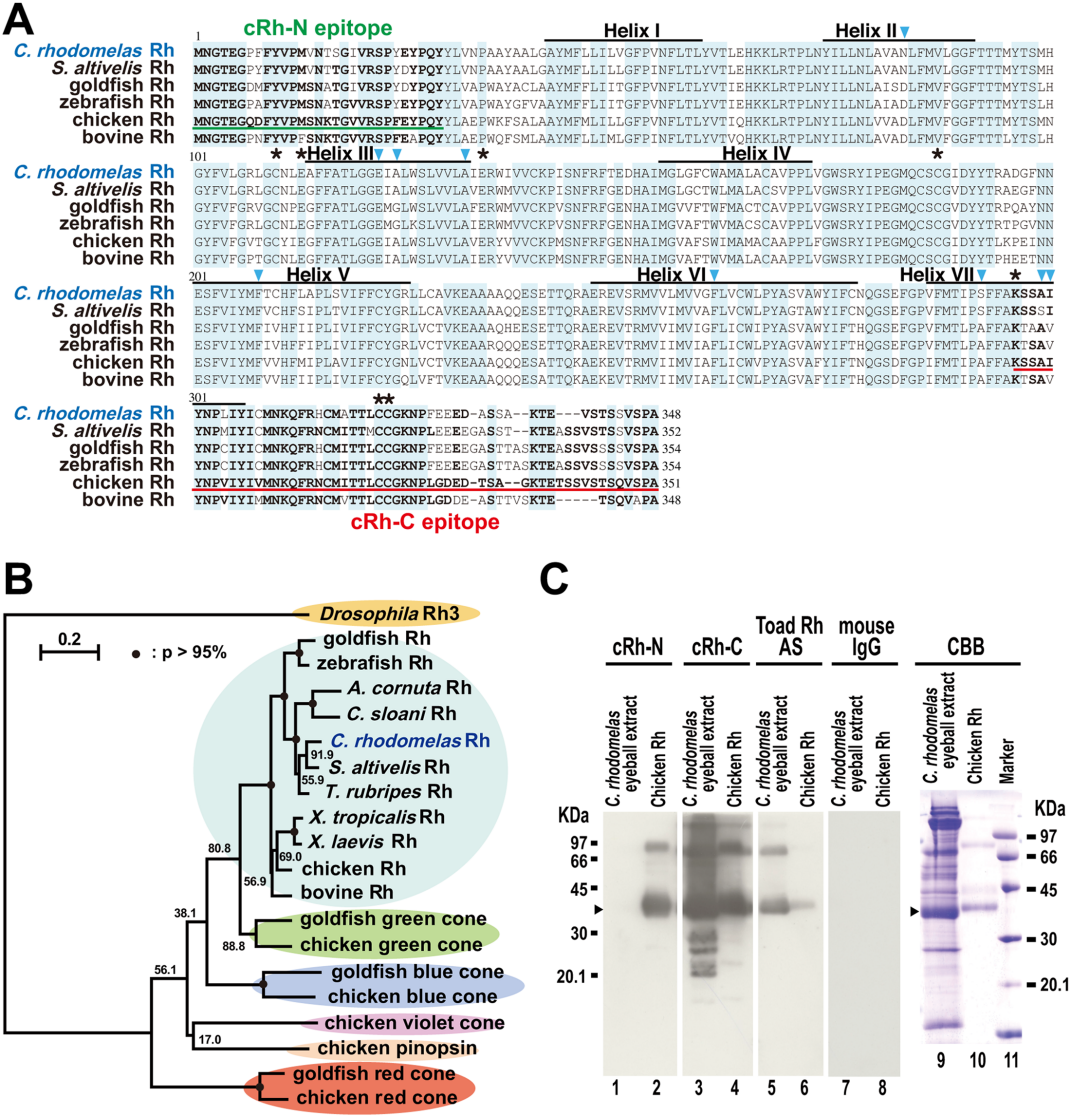
Sequence analysis and immunoblot detection of CrRh. (A) Amino acid sequences of CrRh and the other opsins. Alignment of CrRh and representative members of vertebrate rhodopsins are shown. Amino acid sequences except for CrRh (present study) were obtained from the NCBI Entrez Protein database. *S*. *altivelis* is the deep-sea rockfish lonspine thornyhead [29]. Amino acids sequences were aligned using CLUSTAL W software. Blue-colored boxes indicate conserved regions in all members. Asterisks indicate amino acid positions with functionally important residues. Arrowheads indicate positions for spectral tuning sites based on previous study [16]. Black lines show transmembrane regions. cRh-N epitope is the epitope of polyclonal antibody (cRh-N) which recognizes N-terminal region of chicken rhodopsin (Met_1_-Tyr_29_, [25]). cRh-C epitope is the epitope of polyclonal antibody (cRh-C) which recognizes C-terminal region of chicken rhodopsin (Lys_296_-Ala_351_). Bold letters in the epitope regions denote amino acids identical to those of chicken rhodopsin. (B) A phylogenetic tree of opsin family proteins constructed by Neighbor-Joining method. Amino acid sequences except for *A*. *cornuta* rhodopsin [16], *C*. *sloani* rhodopsin [16], and CrRh (present study) were obtained from the NCBI Entrez Protein database. They were analyzed in the conserved region of the Rh family proteins using CLUSTAL W and NJ plot software (version 2.3). Bootstrap probabilities (p) are represented by closed circles on the nodes (p > 95%) or values near the nodes. Accession numbers for sequences obtained from the NCBI Entrez Protein database are M17718 (*Drosophila* Rh3), L11863 (Goldfish Rh), NM_131084 (Zebrafish Rh), DQ490124 (*Sebastolobus altivelis* Rh), NM_001078631 (*Takifugu rubripes* Rh), NM_001097334 (*Xenopus tropicalis* Rh), NM_001087048 (*Xenopus laevis* Rh), NM_001030606 (Chicken Rh), NM_001014890 (Bovine Rh), L11866 (Goldfish green cone), M92038.1 (Chicken green cone), L11864 (Goldfish blue cone), NM_205517 (Chicken blue cone), NM_205438 (Chicken violet cone), NM_205409 (Chicken pinopsin), L11867 (Goldfish red cone), and NM_205440 (Chicken red cone). (C) Western blot analysis and CBB stain of *C*. *rhodomelas* ocular proteins. For western blotting (lanes 1–8), eyeball extract corresponding to a presumed 0.01 retina (containing 0.0012 ODml of CrRh) of *C*. *rhodomelas* or purified chicken rhodopsin (chicken Rh, 0.5 μg, [27]) was loaded in each lane. For CBB stain (lanes 9–11), the larger amount of the extract (0.03 retina, 0.0036 ODml of CrRh) and purified chicken rhodopsin (3 μg) was loaded. Anti-rhodopsin antibodies used were: cRh-N, anti-N-terminal region of chicken rhodopsin (Met_1_-Tyr_29_, [25]); cRh-C, anti-C-terminal region of chicken rhodopsin (Lys_296_-Ala_351_); Toad Rh AS, anti-toad rhodopsin antiserum [24]. Concentration and dilution of the antibodies were: cRh-N (1000-fold dilution), cRh-C (2.9 ng/ml) and Toad Rh-AS (1000-fold dilution). Control anti-mouse IgG antibody was used at 2.9 ng/ml. Arrowheads indicate signals considered as monomeric form of CrRh.

By using a pair of degenerate primers for rhodopsin, we amplified a part of the cDNA encoding putative CrRh. Then, 5’- and 3’- RACE using specific primers was performed to determine the nucleotide sequences of the rest of the cDNA. Complementary DNA clones covering the entire coding sequence (DDBJ/EMBL/GenBank Acc. No. LC050194) were amplified using a pair of primers designed in the 5’- and 3’- UTRs. Using the Neighbor-Joining method, we constructed a phylogenetic tree of opsins including retinal opsins, pinopsin, insect opsin (as an outgroup), and the deduced amino acid sequence of the putative CrRh (Fig 2A and 2B). The tree showed that CrRh constituted a monophyletic group together with the known vertebrate rhodopsins.

CrRh was composed of 348 amino acids (Fig 2A) including the seven transmembrane α-helical regions of the opsin. A number of functionally important residues characteristic to visual pigments are conserved in CrRh (Fig 2A, asterisks). These conserved residues include (bovine rhodopsin numbering is used for convention): the retinal binding site with Lys296 [30]; Glu113 which forms the counterion to retinylidene Schiff’s base [31]; Cys110 and Cys187 which aid in disulfide bond formation [32]; Cys322 and Cys323 for palmitoylation [33]; and Glu134 which provides a stabilizing negative charge for inactive opsin [34]. Also, the amino acid at position 136 is generally tyrosine in mammals and birds, but tyrosine is replaced by tryptophan in fish [15,35], and this substitution was also seen in CrRh. These features together with the residues for spectral tuning (see below) support the idea that the isolated cDNA encoded CrRh extracted from the eyeball.

### Detection of rhodopsin protein in the eyeball extract

To detect *in vivo* rhodopsin protein expression in the eye, we performed an immunoblot analysis and CBB stain of the *C*. *rhodomelas* eyeball extract (Fig 2C). Two of three kinds of anti-rhodopsin antibodies, cRh-C and ToadRh-AS but not the cRh-N antibody, recognized rhodopsin-like immunoreactivity (Fig 2C, lanes 1, 3, and 5) probably due to the difference in the N-terminal epitope region of cRh-N between cRh and CrRh (Fig 2A).

The molecular mass estimated from the main band mobility (39.0 kDa) agreed well with the molecular mass of CrRh predicted from the cDNA sequence (38.9 kDa, Fig 2A). All three antibodies detected bands for chicken rhodopsin (Fig 2C, lanes 2, 4, and 6) with the estimated and calculated molecular masses of 40.5 kDa and 39.3 kDa, respectively. Bands for rhodopsin dimers were observed at mobilities between 66 kDa and 97 kDa in *C*. *rhodomelas* extract and chicken rhodopsin. The CBB-stained gel showed that rhodopsin was likely the most abundant protein in the *C*. *rhodomelas* eyeball extract (Fig 2C, lane 9).

### Spectroscopic analysis of recombinant CrRh

**Fig 3:**
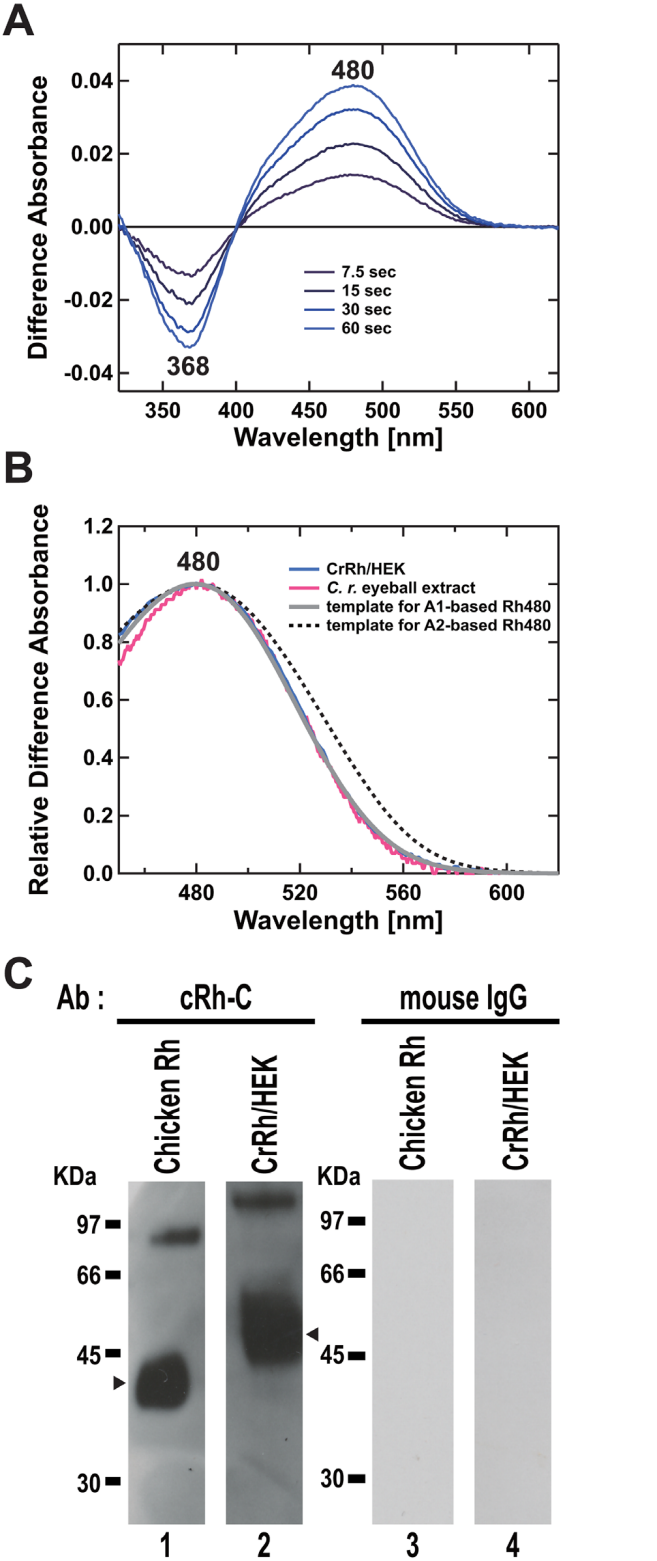
Spectroscopic and immunoblot analyses of recombinant CrRh expressed in HEK293EBNA cells. (A) Spectral changes of recombinant CrRh caused by light irradiation. Proteins were solubilized with buffer P-10 (50 mM HEPES, 10 mM NaCl, 1 mM DTT, 4 μg/mL Aprotinin and 4 μg/mL Leupeptin) containing 1% CHAPS and 0.2 OD 11-*cis* retinal. One molar NH_2_OH was added to the final concentration of 50 mM. Then the sample was sequentially irradiated with green light (501 μW/cm^2^, λ_max_ = 520 nm) for totally 1 min (7.5 sec, 15 sec, 30 sec, and 60 sec). Difference absorbance spectra between those before the first irradiation and after each irradiation are shown (7.5 sec, 15 sec, 30 sec, and 60 sec). (B) Comparison between difference absorption spectrum of eyeball extract and that of recombinant CrRh. The difference absorption spectra of eyeball extract and recombinant CrRh were derived from Fig 1C (O564 2min) and Fig 3A (60 sec), respectively. The spectrum of recombinant CrRh were best fitted with visual pigment template of λ_max_ = 480 nm, which was calculated by the method described in [36]. (C) Western blot analysis of recombinant CrRh. Lanes 1 and 3, purified chicken rhodopsin (cRh; [27]). Lanes 2 and 4, recombinant CrRh proteins expressed in HEK293EBNA. Anti-rhodopsin antibody cRh-C was used. Arrowhead shows a signal considered as a monomeric form of CrRh.

To examine whether the isolated cDNA encodes CrRh detected in the eyeball extract, we expressed recombinant CrRh in HEK293EBNA, and performed spectroscopic and immunoblot analysis (Fig 3). The cells were homogenized, washed to remove soluble proteins, and the resultant membrane fraction was supplemented with 11-*cis* retinal to reconstitute recombinant CrRh in the dark. After NH_2_OH bleaching, the sample was irradiated with green light (501 μW/cm^2^, λ_max_ = 520 nm) until photobleaching was completed (Fig 3A). The difference spectra between the absorption spectra before the first irradiation and after each irradiation showed the difference absorption peak at 480 nm (Fig 3A). The difference spectrum of CrRh (Fig 3B, blue line) agreed well with the difference spectrum obtained by photobleaching of the *C*. *rhodomelas* eyeball extract (Fig 3B, red line) and importantly, these spectra fitted with the template spectrum calculated for A_1_-based Rh480 (rhodopsin with λ_max_ at 480nm)(Fig 3B, grey line) better than that for A_2_-based Rh480 (Fig 3B, dotted line).

The recombinant CrRh was also subjected to immunoblot analysis using anti chicken Rh antibody (Fig 3C). The electrophoretic mobility of the recombinant CrRh (Fig 3C) was smaller than the CrRh in the eyeball extract (Fig 2C) probably due to the mammalian cell-specific posttranslational modification. This result strongly supports that the component with absorption maximum at 480 nm (Fig 1B and 1C) in the eyeball extract is derived from rhodopsin with 11-*cis*-retinal chromophore.

## Discussion

### *C*. *rhodomelas* visual pigment

Comparative analysis of spectral properties of eyeball extract (Fig 1B and 1C) and recombinant protein (Fig 3B) strongly suggested that CrRh possesses the vitamin A_1_-type chromophore to absorb maximally at 480 nm. In the spectroscopic analysis of the ocular CrRh, we used 100 mM NH_2_OH for photic bleaching to completely separate rhodopsin from other retinal-derived compounds, because 100 mM NH_2_OH is known to react with retinal or retinal Schiff’s base or cone opsins but not do with rhodopsin [27]. The addition of 100 mM NH_2_OH to the extract induced a rapid decrease in the 414 nm peak and a concomitant increase in the absorption around 360 nm (Fig 1B, inset). These changes likely indicate the rapid decay of a short-wavelength absorbing substance P_414_ with an increase in absorption around 360 nm. The spectral shape of the UV-absorbing materials with absorption around 360 nm coincides well with that of retinal oximes, but the shape of P_414_ seems to have a narrower bandwidth (~44 nm) than that expected for an opsin with λ_max_ at 414 nm (~83 nm). Like P_414_, the retinal extract peak at around 410 nm has also been observed in some deep-sea fishes [37–39], and it would be interesting to analyze in future whether P_414_ is a cone opsin or not.

We did not detect a decrease in the absorption at longer wavelength regions (> approx. 550 nm) upon the addition of 100 mM NH_2_OH (Fig 1B, inset), indicating the absence of long-wavelength-absorbing cone pigments such as red- or green-sensitive cone pigments in the extract. Normal deep-sea fish (not living near the hydrothermal vents) mostly have only a single visual pigment rhodopsin within their retinas [37–41] with the exception of some species having additional long-wavelength-sensitive pigments of which red sensitivity is thought to facilitate perception of their own long-wavelength bioluminescence [42, 43].

### Spectral tuning of *C*. *rhodomelas* Rh

The absorption maximum of CrRh (480 nm) is blue-shifted in comparison to most shallow water fishes' rhodopsins, yet a common observation in deep-sea marine fishes' [10–13, 38, 40, 41]. Generally, the peak spectral sensitivity of a visual pigment depends on both the chromophore retinal and amino acid sequence. In contrast to most shallow freshwater teleosts that have A_2_-based pigments, the Lake Baikal deepwater cottoids possess the vitamin A_1_-type chromophore [13]. The spectral shift probably arises from amino acid substitutions in the opsin, with rare cases possessing a mixture of vitamin A_1_-based and A_2_-based pigments [44].

In terms of the spectral tuning of CrRh, previous comparative analyses of the spectral properties and amino acid sequences of deep-sea fish rhodopsins have identified nine amino acid replacements [14–16]. All of the nine amino acid combinations are conserved among CrRh and rhodopsins in *A*. *korotneffi*, *C*. *boulengeri* and *H*. *mediteranus* (Table 1), which have λ_max_ at 479–484 nm. These values coincide nicely with the λ_max_ of CrRh (480 nm) and support our present identification of the isolated cDNA that encodes CrRh. In addition, the amino acid sequences of CrRh and *H*. *mediteranus* rhodopsin are not highly conserved in most amino acid sequences (83%), supporting the idea that these nine positions are important determinants for the spectral tuning of rhodopsins in deep-sea fish.

**Table 1:** Amino acid residues at nine sites in rhodopsins of deep-sea fishes.

Order	Species	83	122	124	132	208	261	292	299	300	λ_max_
Scorpaeniformes	*C*. *rhodomelas*	N	E	A	A	F	F	S	A	I	480
	*A*. *korotneffi*	N	E	A	A	F	F	S	A	I	484
	*C*. *boulengeri*	N	E	A	A	F	F	S	A	I	484
	*S*. *altivelis*	N	E	A	A	F	F	S	S	I	483
Beryciformes	*H*. *mediteranus*	N	E	A	A	F	F	S	A	I	479
	*A*. *cornuta*	N	E	A	A	F	F	S	S	I	485
Ophidiformes	*B*. *compresis*	N	E	S	A	F	F	S	A	T	476
	*C*. *laticeps*	N	E	S	A	F	F	S	A	I	468
Gadiformes	*P*. *blennoides*	D	E	A	A	F	F	A	A	I	494
	*C*. *guntheri*	D	V	A	A	F	F	S	A	I	479
Myctophiformes	*D*. *rafinesquei*	D	Q	A	S	F	F	A	A	I	489
	*B*. *suborbitale*	D	Q	G	S	F	F	A	A	I	487
	*L*. *alatus*	N	Q	A	S	F	F	A	A	I	485
	*B*. *indicus*	D	Q	A	S	F	F	A	A	I	489
	*C*. *warmingii*	D	Q	A	S	F	F	A	A	I	487
Aulopiformes	*B*. *ferox*	N	E	S	A	F	F	S	T	L	481
	*B*. *mollis*	N	E	S	A	F	F	S	T	L	479
Stomiiformes	*A*. *aculeatus*	N	Q	A	A	F	F	S	A	L	477
	*A*. *gigas*	N	Q	A	A	F	F	S	A	L	477
	*G*. *elongatum*	N	E	A	A	F	F	S	A	L	482
	*G*. *bathyphilium*	N	E	A	A	F	F	S	A	L	481
	*I*. *ovatus*	N	E	A	A	F	F	S	A	L	489
	*V*. *nimbaria*	N	Q	A	A	F	F	S	A	L	477
	*C*. *sloani*	N	E	G	A	F	F	S	A	L	485
	*C*. *danae*	N	E	A	A	F	F	S	A	L	484
	*S*. *boa*	N	E	G	A	F	F	S	A	L	487
	*I*. *fasciola*	N	E	A	A	F	F	S	A	L	485
	*P*. *guernei*	N	E	G	A	F	F	T	A	L	483
	*A*. *tittmani*	N	E	A	A	F	Y	I	A	L	517
	*M*. *niger*	N	E	A	A	Y	Y	I	A	L	523
Osmeriformes	*A*. *bairdii*	D	Q	V	S	F	F	S	A	I	476
	*C*. *salmonea*	D	Q	A	S	F	F	S	A	I	480

The amino acid sites and λ_max_ values except for *S*. *altivelis* [29] and *C*. *rhodomelas* (present study) were obtained from Hunt *et al*. [14, 16].

### Light sources in the deep oceans

The λ_max_ of CrRh (480 nm, Figs 1C and 3B) agrees well with the residual sunlight in the deep ocean [5, 6] and may be a clue towards understanding the light source for vision in *C*. *rhodomelas*. The general consensus is that deep-sea fish can use light from sources such as downwelling sunlight or bioluminescence.

The congruity of CrRh λ_max_ with downwelling sunlight in the deep sea makes it plausible that *C*. *rhodomelas* might use sunlight as its light source. However, based on the transparency of the sea water [45], intensity of the sunlight at a depth of 1500 m would be at least 23 orders of magnitude lower than that at surface of the sea, so we speculated that *C*. *rhodomelas*, which has laterally-placed eyes, may not be able to detect the low level of sunlight directly at a depth of 1500 m. There is still a possibility that *C*. *rhodomelas* migrate to the shallower position and use sunlight, but hydrothermal vent is a huge supply of viable food, so we inferred that *C*. *rhodomelas* do not need to vertically migrate.

An alternative light source at depths of 1500 m may come from luminous organisms. The emission maxima of luminous organisms in the deep sea are usually similar to residual sunlight [6, 7] and also the λ_max_ determined for CrRh (480 nm). Hence, *C*. *rhodomelas* may recognize the bioluminescence produced by aquatic animals living near the hydrothermal vents. It has been reported that *C*. *rhodomelas* feed on the deep-sea blind shrimp *A*. *longirostris*, which inhabits deep-sea hydrothermal vents with its symbiotic bacteria [23, 46]. Taking into consideration that about ninety-percent of marine organisms can emit light [47], we speculated a possibility that *C*. *rhodomelas* can find prey by receiving bioluminescence emitted by *A*. *longirostris* or its symbiotic bacteria. *C*. *rhodomelas* might also produce its own blue bioluminescence, though there is no supporting evidence to date.

Another possible light source could be the near-infrared light generated from hydrothermal vents [18, 20]. The spectral sensitivity of shrimp rhodopsin, peaking at 500 nm, has a very different spectral curve than the radiation generated from hydrothermal vents, but their tails overlap and hence this shrimp can detect the black-body radiation of the 350°C vents [17, 19]. In the case of CrRh, the λ_max_ is not around 500 nm and is instead blue-shifted, and thus it is unlikely that *C*. *rhodomelas* uses the radiation at hydrothermal vents as a light source.
